# Editorial: New evaluation and management for postoperative cardiopulmonary and renal morbidity and mortality

**DOI:** 10.3389/fmed.2023.1155970

**Published:** 2023-02-28

**Authors:** Ki Tae Jung, Young-Kug Kim

**Affiliations:** ^1^Department of Anesthesiology and Pain Medicine, Chosun University Hospital, College of Medicine and Medical School, Chosun University, Gwangju, Republic of Korea; ^2^Department of Anesthesiology and Pain Medicine, Asan Medical Center, University of Ulsan College of Medicine, Seoul, Republic of Korea

**Keywords:** cardiopulmonary, renal, morbidity, mortality, predictor

Advances in anesthetic and surgical techniques have improved the safety of major operative procedures for patients with unstable medical and surgical conditions (1). However, postoperative morbidity and mortality associated with cardiopulmonary and renal complications are of major concern in patients undergoing surgery (2–6). Early identification and management of postoperative complications are essential during the perioperative period (7–11). This Research Topic of *Frontiers in Medicine* introduces interesting studies for the new evaluation and management for improvement in postoperative morbidity and mortality.

Acute kidney injury (AKI), a sudden loss of kidney function, is a sentinel postoperative complication (12–15). Although the incidence of postoperative AKI varies according to urgency, type of surgery, and diagnostic criteria, it is considered common, with an incidence of up to 39% (16). For surgical patients, early detection to prevent and intervene AKI is crucial, given the associated long-term risk of morbidity and mortality (17–19). Novel AKI biomarkers have been heavily researched, and their adoption to predict AKI has been advocated for early intervention in high-risk patients (18–20). The pathophysiology of AKI is complex and multifactorial; therefore, biomarkers tailored to the underlying risks of the individual patient are needed to provide personalized postoperative management. Wu et al. explored the predictive value of glycosylated hemoglobin (hemoglobin A1c, HbA1c) for postoperative AKI in non-cardiac surgery. Elevation of pre-operative HbA1c was an independent risk factor for AKI (OR comparing top to bottom quintiles 5.02, 95% CI: 1.90–13.24, *P* < 0.001 for trend; OR per percentage point increment in HbA1c 1.20, 95% CI: 1.07–1.33). They suggested that elevated HbA1c is associated with arteriosclerosis through glycosylation changes in the mesangial matrix, contributing to postoperative AKI. These findings support the research showing that aberrant glucose metabolism is associated with the pathogenesis of AKI (21). Therefore, HbA1c may be a new AKI predictive value in patients undergoing non-cardiac surgery.

Patients undergoing cardiac surgery are at particular risk for AKI, with an incidence of up to 30% (22). The pathophysiology of AKI is also complex and multifactorial, including fluctuation of hemodynamics and perturbation in inflammatory mechanisms (22). In the case of major cardiac surgery of patients with Stanford type A aortic dissection, serum myoglobin can improve postoperative AKI risk classification. Yang et al. studied the correlation between serum myoglobin and AKI after total aortic arch replacement combined with frozen elephant trunk implantation. Patients with AKI showed a higher level of perioperative serum myoglobin than those without. Moreover, levels of serum myoglobin before and on the 1st day after surgery were associated with severe AKI [OR = 1.58 (95% CI: 1.26–1.95), *P* < 0.001; OR = 3.47 (95% CI: 2.27–5.29), *P* < 0.001]. They suggested that rhabdomyolysis could be a mechanism of AKI after total aortic arch replacement; thus, elevated serum myoglobin level could be a good predictor of AKI in patients with Stanford type A aortic dissection.

Wang et al. also studied the protective effects of N-acetylcysteine (N-AC) on post-resuscitation AKI. They used rat cardiac arrest models to evaluate renal function, pathologic changes, oxidative stress, inflammatory responses, and apoptosis. N-AC inhibited post-resuscitation AKI by upregulation of the nuclear factor erythroid-2-related factor 2 (Nrf2)/heme oxygenase-1 (HO-1) pathway, which is associated with anti-inflammatory, anti-oxidative, and anti-apoptotic effects after ischemia/reperfusion injury. They concluded that N-AC, a common clinical agent, may potentially improve the outcome of cardiac arrest patients through renal protection *via* activation of the Nrf2/HO-1 pathway.

Dexmedetomidine, a popular drug in critical care, is a highly selective α-2 adrenoceptor agonist with properties including sedation, analgesia, and anxiolysis (23). Dexmedetomidine provides adequate sedation to prevent delirium and decreases the duration of mechanical ventilation for critically ill patients (23, 24). Sun et al. tested the effect of low-dose dexmedetomidine infusion (0.1–0.2 μg/kg/h) on nighttime sleep quality in postoperative intensive care unit patients with invasive mechanical ventilation. Administration of low-dose dexmedetomidine for up to 72 h in patients with required invasive mechanical ventilation after non-cardiac surgery did not significantly improve the sleep quality pattern measured using the Richards–Campbell Sleep Questionnaire [scores: 0–100, with a higher score indicating better quality; overall subjective sleep quality, median 61 (interquartile 27, 79) vs. 52 (20, 66) with placebo; median difference 8, 95% CI: −2, 22; *P* = 0.120]. Despite statistical insignificance, there were trends of improvement in total sleep time [median difference 54 min (95% CI: −4 min, 120 min); *P* = 0.061], sleep efficiency [median difference 10.0% (95% CI: −0.8%, 22.3%); *P* = 0.060], percentage of stage N1 sleep [median difference −3.9% (95% CI: −11.8%, 0.5%); *P* = 0.090], percentage of stage N3 sleep [median difference 0.0% (95% CI: 0.0%, 0.4%); *P* = 0.057], and arousal index [median difference −0.9 (95% CI: −2.2, 0.1); *P* = 0.091]. They concluded that the underpowered sample size led to differences without statistical significance and suggested a large, randomized trial to investigate the effect of low-dose dexmedetomidine on sleep quality in this patient population.

This editorial overviewed new evaluation and management methods for postoperative morbidity and mortality. Appropriate and careful perioperative risk stratification and management are crucial to improve perioperative anesthetic and surgical outcomes. However, further studies are needed to clarify this association given the limited number of publications on this Research Topic.

## Author contributions

This editorial was prepared jointly by KJ and Y-KK. Both authors contributed to the article and approved the submitted version.
